# It’s Not Always Occam's Razor – The Case of a Young Man with Subaortic Membrane with Superimposed Pulmonary Thromboembolism and Left Main Coronary Artery Disease

**DOI:** 10.7759/cureus.5850

**Published:** 2019-10-06

**Authors:** Hunaina Shahab, Abdul Baqi, Yusra Askari, Qurrat Ul Ain Aqeel, Kumail Khandwala

**Affiliations:** 1 Cardiology, Aga Khan University Hospital, Karachi, PAK; 2 Medicine, Aga Khan University Hospital, Karachi, PAK; 3 Medicine, Aga Khan University Hospital, Karachi, PAK; 4 Radiology, Aga Khan University Hospital, Karachi, PAK

**Keywords:** sub-aortic membrane, pulmonary hypertension, chronic pulmonary thromboembolism

## Abstract

Subaortic membrane (SAM) is a discrete fibromuscular structure which causes left ventricular outflow tract obstruction and leads to the symptomatology of valvular aortic stenosis. It is known to be associated with other congenital cardiac defects in around 30% of cases. However, it has not been associated with chronic pulmonary thromboembolism in the past. We present a case of a middle-aged Pakistani man who presented with dyspnea and hemoptysis. He was found to have a SAM and severe pulmonary hypertension on transthoracic echocardiogram. A coronary angiogram revealed non-obstructive left main coronary artery disease. A computed tomography (CT) scan chest was done to evaluate the cause of severe pulmonary hypertension unexplained by SAM which revealed chronic pulmonary thromboembolism. Surgical resection was deferred due to high risk. Hence, he was kept on anticoagulation for pulmonary thromboembolism, and aspirin and a statin for non-obstructive coronary artery disease. Over the course of two months, his symptoms improved. This case highlights the importance of evaluating different causes of pulmonary hypertension in patients with SAM.

## Introduction

Subaortic membrane (SAM) is a fibromuscular ridge which causes obstruction of the left ventricular outflow tract (LVOT) and the aftermath is similar to valvular aortic stenosis as well destruction of the aortic valve due to disrupted dynamics of the LVOT, thus causing aortic regurgitation [1,2]. The etiology is an amalgamation of an inherent genetic predisposition along with geometric and anatomic discrepancies in the LVOT which causes turbulence in blood flow, thus damaging the endothelium and causing fibrin deposition [3]. A literature review describes the association of SAM with coronary artery disease [4]. However, to the best of our knowledge, there has been no prior documentation of the association of pulmonary thromboembolism with discrete SAM. We describe the case of a middle-aged man who presented with dyspnea and hemoptysis and was found to have SAM with superimposed non-obstructive coronary artery disease along with severe pulmonary artery hypertension secondary to chronic pulmonary thromboembolism.

## Case presentation

**Figure 1:**
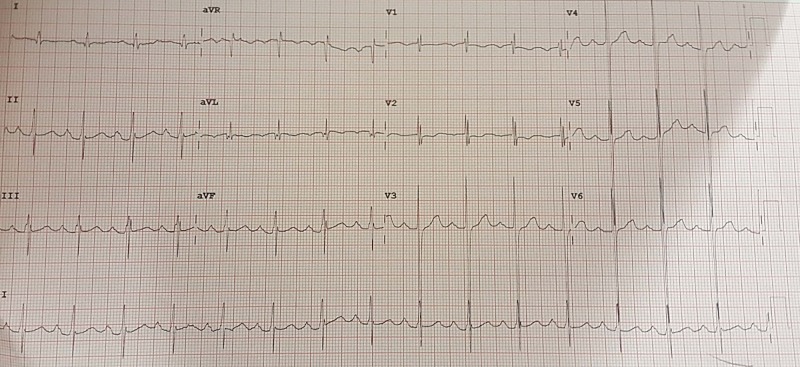
Electrocardiogram An electrocardiogram showed normal sinus rhythm at the rate of 98 beats per minute, leftward axis and rSR’ pattern in lead V1.

A 42-year-old man from Pakistan presented with a history of four episodes of hemoptysis for one month prior to presentation. Each of the episodes had two to three teaspoons of fresh blood mixed with slightly yellowish sputum. He also complained of dyspnea on climbing more than one flight of stairs, persistently for the past 20 years which he attributed to his asthma and did not undergo any specific investigations. The dyspnea on exertion had started to worsen for the last two months. He denied fever, orthopnea, paroxysmal nocturnal dyspnea, chest pain, palpitations, syncope, weight loss or exposure to tuberculosis. His past medical history was only significant for asthma. He had no addictions, no use of anorexigens and was not aware of any drug or food allergies. Family history was not significant. For these complaints, he visited the cardiology clinic. On physical examination, he had a heart rate of 100 beats per minute, blood pressure of 142/69 mmHg, a temperature of 37.4 degrees Celsius and was maintaining an oxygen saturation of 97% on room air. There was no visible rash, hyperpigmentation, clubbing or cyanosis. On chest examination, he had no deformities, no visible scars or pulsations. On auscultation, he had normal vesicular breathing bilaterally. Cardiac auscultation revealed a normal S1, soft S2 with a grade 3/6 ejection systolic murmur at the aortic area radiating to the carotids and a grade 2/6 pan-systolic murmur at the tricuspid area. His abdomen was soft and non-tender with no hepatosplenomegaly. An electrocardiogram was done which is shown in Figure 1.

**Figure 2:**
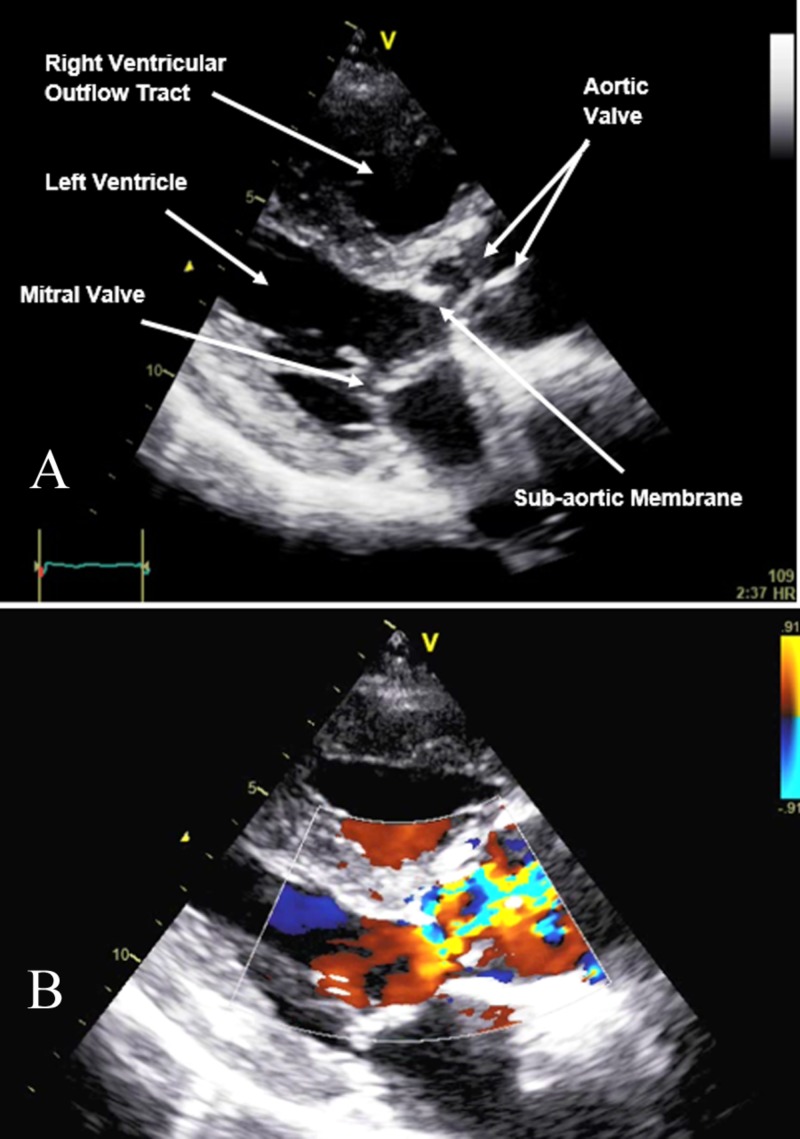
Transthoracic Echocardiogram A: Parasternal long axis of transthoracic echocardiogram showing subaortic membrane. Other normal structures seen are aortic valve, mitral valve, left ventricle and right ventricular outflow tract. B: Parasternal long axis of transthoracic echocardiogram with Color Doppler applied at the level of the aortic valve revealing turbulence below the level of the aortic valve.

A transthoracic echocardiogram was subsequently done which showed a visually estimated ejection fraction of approximately 60%. The aortic valve was thickened, calcified with redundant relatively immobile left and non-coronary cusps with mild (eccentric) aortic regurgitation but without any visual evidence of significant stenosis. SAM was noted, as shown in Figure 2, with a peak pressure gradient of 58 mmHg and a mean pressure gradient of 44 mmHg. Estimated pulmonary artery systolic pressure was approximately 90 mmHg. Inferior vena cava was dilated with loss of inspiratory collapse. The pulmonary artery was dilated with a mildly dilated aortic root. Right ventricular systolic function was normal. There was grade 1 diastolic dysfunction with E/E’ of 22. 

**Figure 3:**
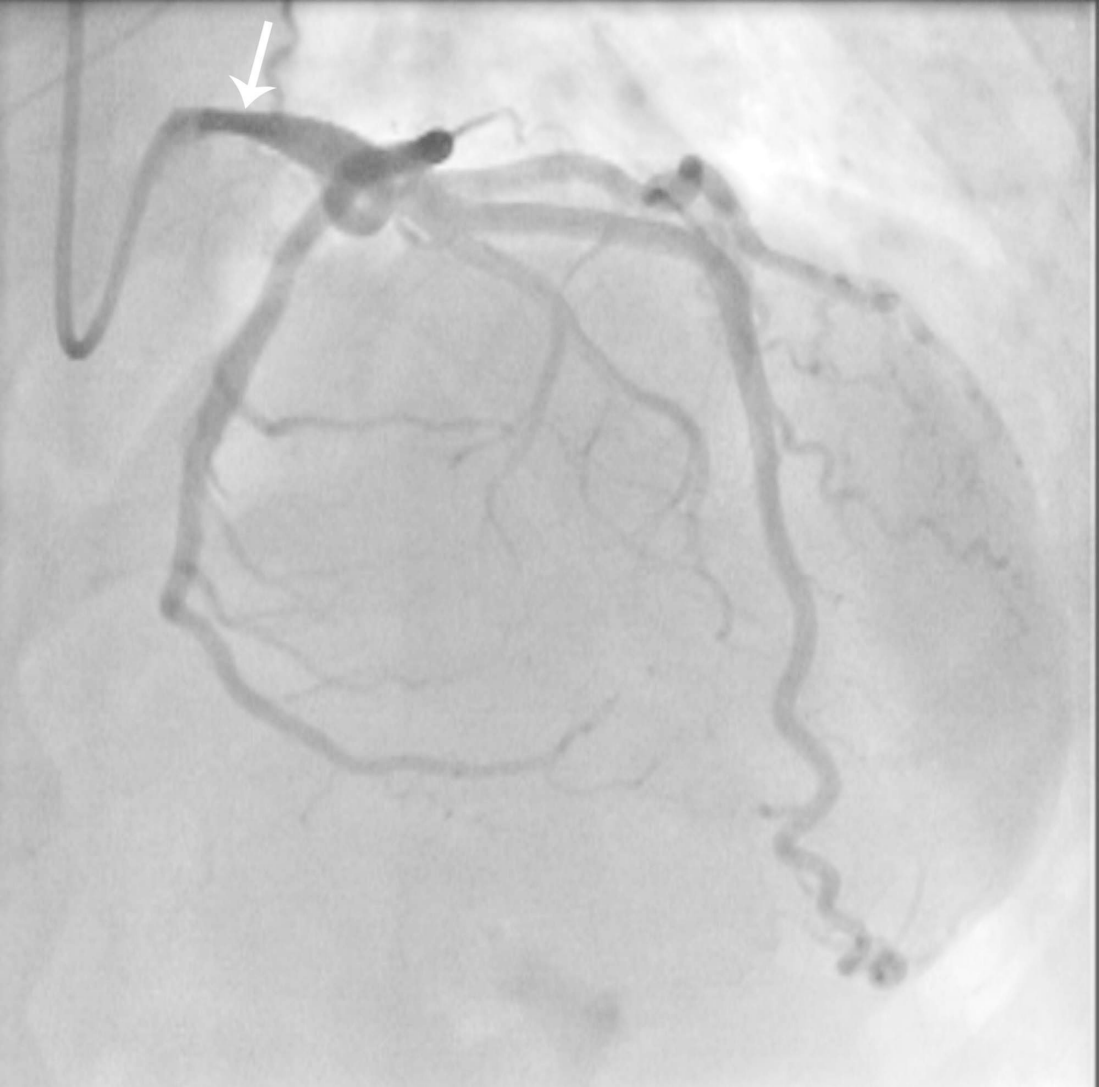
Coronary Angiogram Right anterior oblique cranial (RAO cranial) fluoroscopic view showing the engaged catheter in the left main artery branching into the left anterior descending artery, giving off a large diagonal branch, and the left circumflex artery, giving rise to the obtuse marginal branches. Proximal left main coronary artery shows a lesion of 50% (arrow).

The patient was then referred for a coronary angiogram which showed normal coronaries. However, it showed a 50% stenotic lesion in the left main coronary artery as shown in Figure 3. It was decided to proceed for fractional flow reserve (FFR) measurement to assess for the significance of left main disease. The FFR of the left main coronary artery with adenosine hyperemia was 0.96.

**Figure 4:**
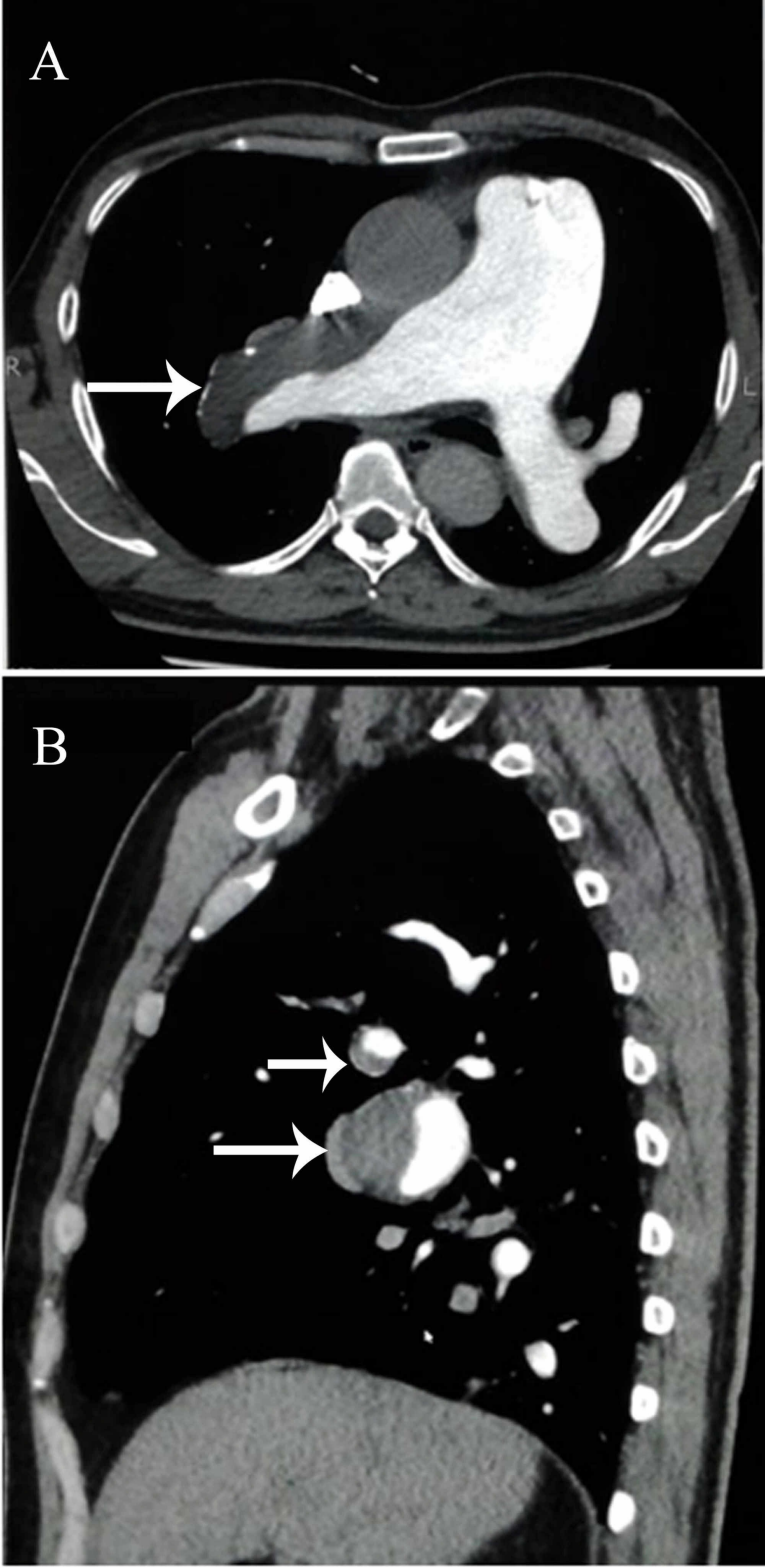
Computed Tomography Scan Chest Axial section (A) and sagittal section (B) through the thorax: An eccentric filling defect is seen in right main pulmonary artery also involving the segmental branches of lower lobe suggestive of a thrombus (arrows). There is associated dilatation of the main pulmonary trunk.

The high pulmonary artery pressures could not be explained by the SAM. Serological workup for collagen vascular disease, vasculitis and human immunodeficiency virus (HIV) was negative. It was therefore decided to get a computed tomography (CT) scan of the chest to rule out pulmonary embolism. The chest CT scan showed an eccentric thrombus occupying the right main pulmonary artery associated with peripheral calcification as shown in Figure 4. This was likely secondary to chronic pulmonary thromboembolism. The thrombus appeared to be extending into the segmental branches of the right pulmonary artery. It resulted in significant proximal dilatation of the pulmonary trunk and left main pulmonary artery. The appearances were suggestive of pulmonary hypertension with peripheral pruning of vessels at the right lower lung zone. Areas of mosaic perfusion and ground-glass haze predominantly involving lower lobe of the right lung were present which could be secondary to hemorrhage. Hypercoagulability workup which included Protein C and S, anti-thrombin III and activated protein C resistance was conducted and was within normal limits.

The patient was immediately referred to cardiothoracic surgery for resection of the subaortic membrane and pulmonary artery thrombectomy. However, due to the severe pulmonary artery hypertension, the patient needed a right ventricular assist device, the facilities of which were unavailable at our center. Therefore it was decided to manage the patient conservatively with anti-coagulation for the pulmonary embolism, and with statin and low dose aspirin for the non-obstructive coronary artery disease. On a two month follow-up, the patient reported an improvement in dyspnea and the hemoptysis had resolved.

## Discussion

Subaortic stenosis is a rare congenital cardiac anomaly, accounting for about 1-2% of all of them, with SAM representing about 70% of the scale [5,6]. About 30% of patients with SAM have associated congenital heart defects including ventricular septal defect, coarctation or interrupted aortic arch, bicuspid aortic valve, supra-valvular mitral stenosis and persistent left superior vena cava [5]. In 2005, a case reported the presence of an obstructive left anterior descending (LAD) artery along with a SAM which was successfully treated by excision of the membrane and anastomosis of the left internal mammary artery to the LAD [4]. A case of a 20-year-old patient with SAM had been reported with ruptured sinus of Valsalva and infective endocarditis which led to thromboembolic sequelae [1]. However, to the best of our knowledge, the spectrum of SAM with chronic pulmonary thromboembolism along with non-significant left main coronary artery disease in a middle-aged-man has not been reported.

Our patient had SAM and non-obstructive coronary artery disease. However, both the pathologies were unable to account for such high pulmonary artery systolic pressures, which led to the CT scan of the chest showing chronic pulmonary thromboembolism. The workup for the major causes of chronic pulmonary thromboembolism in our patient was negative, which leads to the possibility of its rare association with SAM.

The appropriate management of subaortic stenosis is trans-aortic surgical resection with low operative mortality rates and favorable immediate postoperative results [7]. The existence of severe pulmonary hypertension in our patient hindered his surgical management and therefore conservative management was opted for. It is important that all patients who present with SAM and pulmonary hypertension must be worked up thoroughly to exclude other causes before referring them for surgical excision.

## Conclusions

Subaortic membrane is a rare congenital cardiac defect which is isolated in around 70% of cases. We report an unusual association of chronic pulmonary thromboembolism and non-obstructive coronary artery disease with a subaortic membrane. It is important to rule out other causes of pulmonary hypertension when it presents with subaortic stenosis.
